# Graphene-Tuned, Tightly Coupled Hybrid Plasmonic Meta-Atoms

**DOI:** 10.3390/nano14080713

**Published:** 2024-04-19

**Authors:** Kai Chen, Ke Li, Yiming Wang, Zihao Zhang, Yanpeng Shi, Aimin Song, Yifei Zhang

**Affiliations:** 1 Shandong Technology Center of Nanodevices and Integration, School of Integrated Circuits, Shandong University, Jinan 250100, China; 202212306@mail.sdu.edu.cn (K.C.);; 2 Key Laboratory for Physical Electronics and Devices of the Ministry of Education & Shaanxi Key Laboratory of Information Photonic Technique, School of Electronic Science and Engineering, Faculty of Electronic and Information Engineering, Xi’an Jiaotong University, Xi’an 710049, China; like_xian@stu.xjtu.edu.cn; 3 Institute of Nanoscience and Applications, Southern University of Science and Technology, Shenzhen 518055, China; 4 Department of Electrical and Electronic Engineering, University of Manchester, Manchester M13 9PL, UK

**Keywords:** tightly coupled meta-atom, graphene, terahertz, tightly coupled antenna array, plasmonic

## Abstract

Tightly coupled meta-atoms (TCMAs) are densely packed metamaterials with unnatural refractive indexes. Actively modulated TCMAs with tunable optical properties have found many applications in beam shaping, holography, and enhanced light–matter interactions. Typically, TCMAs are studied in the classic Bloch theory. Here, tightly coupled H-shaped meta-atoms are proposed with an ultra-high permittivity of ~6000, and their active modulation with graphene is designed by using the tightly coupled dipole array (TCDA) theory. The H-shaped meta-atoms are used as dipole arms, and the graphene strips function as the dipole loads. By tuning the chemical potential of graphene, the resonant amplitude, frequency, and permittivity are dynamically modulated. The simulations indicate that the real and imaginary parts of permittivity change from 6854 to 1522 and from 7356 to 2870, respectively. The experimental validation demonstrates a modulation depth of 11.6% in the resonant frequency, i.e., from 219.4 to 195 GHz, and a substantial 52.5% modulation depth in transmittance under a bias voltage of less than 1.5 V.

## 1. Introduction

Tightly coupled meta-atoms (TCMAs) with densely packed pixels have emerged as a rising frontier in the metamaterial community, offering enhanced light–matter interactions and advanced optical functionalities for lenses and beam shaping [1,2,3]. Differing from conventional metamaterials (MTMs), TCMAs derive their optical potency from the interplays between neighboring pixels, but not from each individual pixel [4]. These strong mutual couplings in subwavelength scales engender compelling properties, such as ultra-high refractive indexes, exceptional transmission, and non-dispersive optical activity [5,6]. To optimize the utilization of TCMA devices, it is imperative to deploy actively manipulative technologies capable of modulating their optical responses and emulating fundamental physical effects through integrated devices and materials. Recent studies have delved into active TCMAs, aiming to achieve dynamic optical properties using different stimuli, such as vanadium oxide (VO_2_) and graphene [7,8]. Graphene, which is a single layer of carbon atoms with a thickness of approximately 0.34 nm, is a promising modulation material due to its unique properties, such as ductility, high electron mobility, excellent thermal stability, good optical transparency, and tunable electric conductivity under an electric field [9]. With the integration of graphene, physical properties ranging from fundamental parameters like amplitude, resonant frequency, and phase modulation to more intricate phenomena such as birefringence, chirality, and active molecularization have been investigated in active TCMAs [1]. In 2013, Lee et al. demonstrated the intensity modulation of terahertz waves by integrating gated single-layer graphene with a non-resonant meta-atom structure. This approach showcases a frequency-insensitive response and facilitates extensive modulation across a broad frequency range from 0.3 to 2.3 THz [10]. Kang et al. found that adjusting very small distances between unit resonators in TCMAs can greatly impact their wavelength scaling in 2018 [11]. In the same year, Jung et al. refined the resonant properties of terahertz TCMAs by controlling electrical connections among metal unit structures using a technique termed “molecularization” [12]. Lee et al. designed a single-layer terahertz metasurface utilizing tightly coupled elementary resonators in 2019. This metasurface serves as an efficient terahertz waveplate, boasting the ability to induce phase retardation of up to 180° while operating at a tunable frequency [13]. Jung et al. reported on the integration of graphene bridges between TCMAs, showcasing the atomic-level modulation of molecularization, resonant frequencies, and phase in 2022 [14]. Park et al. investigated electrically tunable terahertz (THz) wave retarders with graphene metasurfaces, achieving retardations between 15° and 81° for two orthogonal polarization states in 2023 [15]. 

Though many tunable metal–graphene meta-atom structures have been reported in the aforementioned works in the terahertz range and have led to various applications in telecommunication, imaging devices, and ultrasensitive sensors, their modulation mechanisms are investigated in periodic pixels with the classic Bloch theory. As an analog in the antenna community, tightly coupled dipole array (TCDA) has gained great interest in the last twenty years [16]. The strong mutual coupling between dipoles “connects” the dipoles, and thus the array functions, as Wheeler’s current sheets for bandwidth improvement [17]. Among the reported TCMAs, I-shaped resonators represent a classic structural archetype achieving remarkably high refractive indexes and permittivity levels, which are capacitively connected due to the tight coupling between neighboring pixels [6]. In this respect, I-shaped TCMAs may be considered as TCDAs with infinite load resistors.

**Figure 1:**
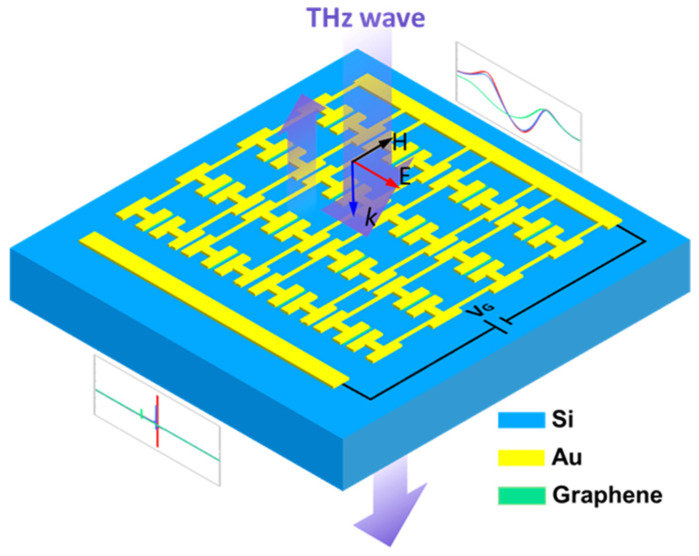
Schematic of graphene-tuned tightly coupled meta-atoms which perform with ultra-high permittivity and achieve deep modulation in transmission, resonant frequency, and refractive index.

In this paper, H-shaped TCMAs with high permittivity of 5775 are proposed at THz frequencies, and TCDA theory is utilized to explain the graphene modulation principle of TCMAs, as shown in Figure 1. Compared to the conventional I-shaped structure, the dipole arms of the H-shaped metastructures are enlarged, which significantly expands the light–matter interaction zone, thereby enhancing the coupling between meta-atom units. To investigate the mutual coupling effect, various structural parameters like the dipole arm length, periodicity, and gap area between meta-atom units are finely adjusted to reveal the inherent mechanisms of TCMAs. By integrating graphene strips as the plasmonic dipole loads, the resonant frequency and amplitude are actively tuned by sweeping the graphene resistance. The simulated modulation depths of resonant frequency and transmittance are 13.2% and 100%, respectively, which correspond to a modulation depth of permittivity up to 77.8%. The experimental results show a modulation depth of 11.6% for the resonant frequency and a modulation depth of 52.5% for the transmittance. This advancement holds promise for applications in sensing, cloaking, and THz signal processing.

## 2. Results and Discussion

### 2.1. Theory

**Figure 2:**
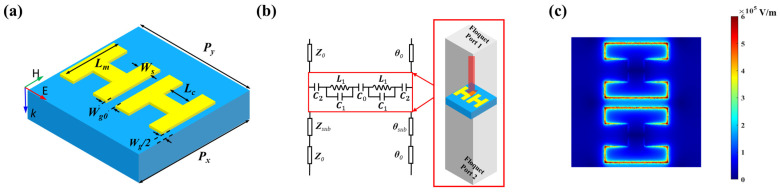
(**a**) The structural configuration of the TCMAs, where key geometric parameters are defined as follows: *P_x_* = 250 μm, *W_s_* = 30 μm, and *L_c_* = 45 μm. (**b**) The ECM of the proposed TCMAs, with *Z*_0_ set at 377 Ω. (**c**) Electric field diagram of the proposed H-shaped TCMAs at 261 GHz.

The model of the proposed H-shaped TCMAs on a silicon substrate is shown in Figure 2a. The period along the *x* and *y* directions is denoted as *P_x_* and *P_y_*, respectively. *L_m_* is the length of the H-shape, and *W_g_*_0_ represents the gap within the TCMAs. The specific structural parameters are elucidated in the caption of Figure 2. THz waves are incident perpendicularly to the surface of the TCMAs with their electric field polarized vertically to the gap between the H-shaped units. To delve deeper into the proposed structures, transmission lines (TLs) and an equivalent circuit model (ECM) are employed, as illustrated in Figure 2b. In this model, *Z*_0_ and *θ*_0_ denote the wave impedance and electric length of free space, and *Z_sub_* and *θ_sub_* are the characteristic impedance and electric length in the silicon. In the ECM of TCMA, *C*_0_ signifies the coupling capacitance between adjacent H-shaped structures, while *C*_1_ and *L*_1_ represent the effective capacitance and inductance of an H-shaped meta-atom unit, respectively. *C*_2_ represents the mutual coupling capacitance between TCMAs. The complex permittivity can be extracted by the following the calculation formula:

(1) $$z=\pm \sqrt{\frac{{\left(1+\tau \right)}^{2}-T^{2}}{{\left(1-\tau \right)}^{2}-{\;T}^{2}}},$$

(2) $$x=\frac{T}{1-\tau \left(\frac{z-1}{z+1}\right)},$$

(3) $$\tilde{n}=\pm \;i\frac{log(x)}{kd},$$

(4) $$\varepsilon =\frac{\tilde{n}}{z},$$

where τ and *T* represent the reflection and transmission coefficient, respectively. Here, *z* and $\tilde{n}$ represent the effective metamaterial impedance and complex refractive index of TCMAs, respectively. *k* is the wave number, and *d* is the effective height of TCMAs [18]. Notably, to obtain the complex permittivity *ε* of the structure, the signs of *z* and $\tilde{n}\;$ should satisfy the following conditions:

(5) z=R+jX, R ≥ 0,

(6) $$\tilde{n}=n+i\kappa ,\;\kappa \geq 0.$$

where the real part *R* is the resistance and the imaginary part *X* is the reactance. The real part *n* is the refractive index, while the imaginary part *κ* is called the optical extinction coefficient. The imaginary units are denoted by *j* and *i*.

**Figure 3:**
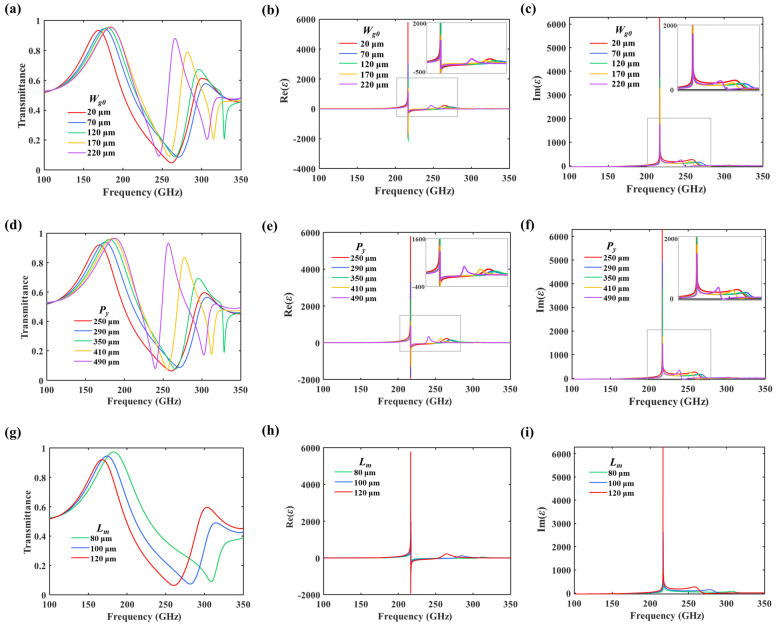
(**a**–**c**) Frequency spectrum of transmittance and the permittivity, respectively, with varying structural parameters *W_g_*_0_. (**d**–**f**) Frequency spectrum of transmittance and the permittivity, respectively, with varying structural parameters *P_y_*. (**g**–**i**) Frequency spectrum of transmittance and the permittivity, respectively, with varying structural parameters *L_m_*.

As mentioned at the end of the introduction, the arm length and periodicity will influence the mutual coupling between TCMAs. In other words, the gap *W_g_*_0_ influences *C*_0_ between two H-shaped units, and *P_y_* and *L_m_* significantly influence *C*_2_ between the neighboring atoms. Therefore, parametric sweeps are conducted to reveal the mechanism of TCMAs. In the simulation, the width of the gap *W_g_*_0_ is swept from 20 to 220 μm to observe the coupling between two H-shaped units. In addition, parameter *P_y_* is swept from 250 to 490 μm under a fixed *L_m_* equal to 120 μm, while *L_m_* ranges from 80 to 120 μm with an increment of 20 μm under a selected *P_y_* of 250 μm. As depicted in Figure 3a–c, an increase in the parameter *W_g_*_0_ leads to a reduction in the coupling within the structural units, resulting in a narrower transmittance bandwidth and lower permittivity. Similarly, the coupling between structural units decreases as *P_y_* increases, causing the low-frequency transmittance bandwidth to narrow and the permittivity to decrease, as shown in Figure 3d–f. Additionally, the arm length *L_m_* of the H-shaped structure relates to the coupling capacitor between adjacent meta-atoms. As *L_m_* rises, the coupling capacitance and resonant strength increase, leading to a widening transmittance bandwidth and increasing permittivity; see Figure 3g–i. In the given simulations, *W_g_*_0_ = 20 μm, *P_y_* = 250 μm, and *L_m_* = 120 μm are selected as model parameters to provide strong coupling and high permittivity. At the resonant frequency of 216 GHz, the peak values of the real and imaginary parts of permittivity are observed as 5775 and 6286, respectively, obtaining ultra-high permittivity in this work. Figure 2c shows the electric field distribution at the resonant frequency. The electric field assembly inside the capacitor and between meta-atom units effectively connects the dipoles. Therefore, the H-shaped TCMAs can be regarded as TCDAs with infinite load resistors.

### 2.2. Graphene-Tuned TCMAs

**Figure 4:**
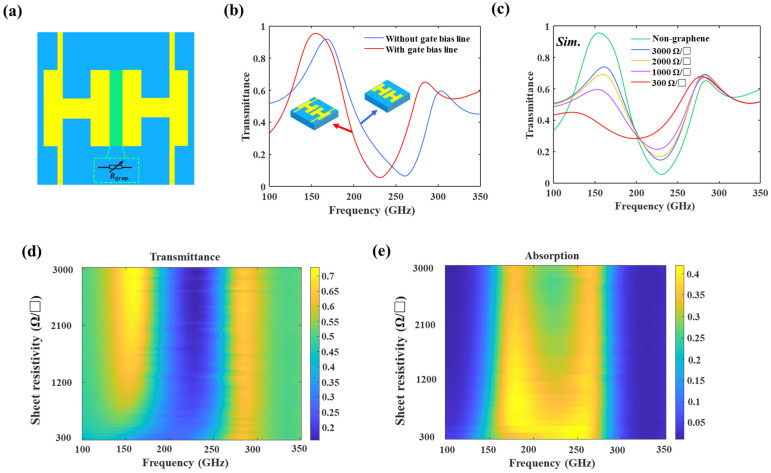
(**a**) TCMAs integrated with graphene. (**b**) Comparison of transmittance with and without the gate bias lines. (**c**) Simulated transmittance without the graphene and with graphene as *ρ_s_* sweeps from 3000 to 300 Ω/□ (the corresponding resistor is from 500 to 50 Ω). The color plot of transmittance (**d**) and absorption (**e**), respectively.

In the TCDA design, 50-Ω transmission lines are typically preferred to feed the dipole elements, where impedance matches should be carefully considered to broaden the bandwidth [19]. Similarly, graphene strips are designed between two H-shaped dipoles as effective loads in our work. By tuning the chemical potential of graphene, the impedance match between the load and dipoles is changed, leading to active modulation. For the convenience of applying voltages on graphene, gate bias lines have to be added to H-shaped TCMAs in the experiment, which is beneficial for the coupling capacitance *C*_2_. In the ECM, the graphene can be equivalent to a tunable resistor *R_grap_*, as shown in Figure 4a. As can be seen from Figure 4b, adding bias lines red-shifts the resonant frequency without significantly changing the permittivity. 

In our simulation, the graphene model is set as a conductive sheet [20]. In fact, the transferred chemical vapor deposition graphene usually has higher sheet resistance than the ideal values derived from the Kubo formula due to the defects. According to the direct-current (DC) measurements of a graphene field-effect transistor, the experimental sheet resistance is from 3000 to 300 Ω/□, which corresponds to a load with resistance from 500 to 50 Ω. The resultant simulated transmittance characteristics are depicted in Figure 4c. A transmittance dip of 0.14 is found at 229.3 GHz with *ρ_s_* = 3000 Ω/□, and that of 0.28 is observed at 199 GHz with *ρ_s_* = 300 Ω/□. Consequently, the modulation depths of resonant frequency and transmittance amplitude approach 13.2% and 100%, respectively. Figure 4d presents a colormap of transmittance as a function of frequency and graphene resistance values. Notably, as the resistance of graphene decreases, the resonant frequency of the structure red-shifts, while the transmittance amplitude increases. The absorption characteristics of the structure across various resistivities of graphene are illustrated in Figure 4e. Remarkably, a decrease in resistance leads to a widening of the absorption bandwidth and an attenuating resonance, which is the key function of TCDAs.

**Figure 5:**
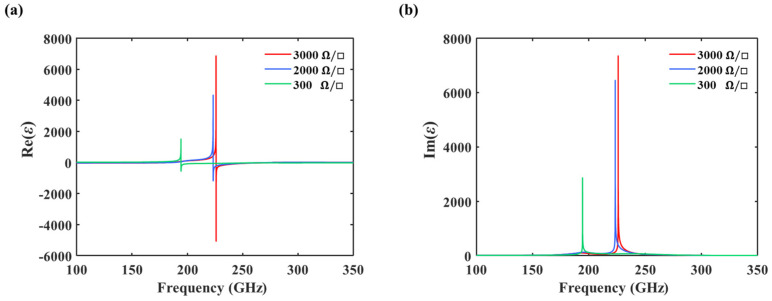
(**a**,**b**) A comparison of the real and imaginary parts of permittivity with the graphene sheet resistance of 3000, 2000, and 300 Ω/□.

Figure 5a,b provide a comparative analysis of the extracted real and imaginary components of permittivity of the TCDAs with sheet resistances ρ_s_ = 3000, 2000, and 300 Ω/□. The peak value of the real part of permittivity is 6854 at 226 GHz at ρ_s_ = 3000 Ω/□ and 4347 at 223 GHz at ρ_s_ = 2000 Ω/□ and decreases to 1522 at 194 GHz at ρ_s_ = 300 Ω/□. The corresponding modulation depth is 77.8%. On the other hand, the peak value of the imaginary part is 7356 at ρ_s_ = 3000 Ω/□ and 6456 at ρ_s_ = 2000 Ω/□, respectively, and decreases to 2870 at ρ_s_ = 300 Ω/□, revealing a modulation depth of up to 61% for the imaginary part of permittivity. The frequency modulation is from 226 to 194 GHz, resulting in a modulation depth of 14.2%.

**Figure 6:**
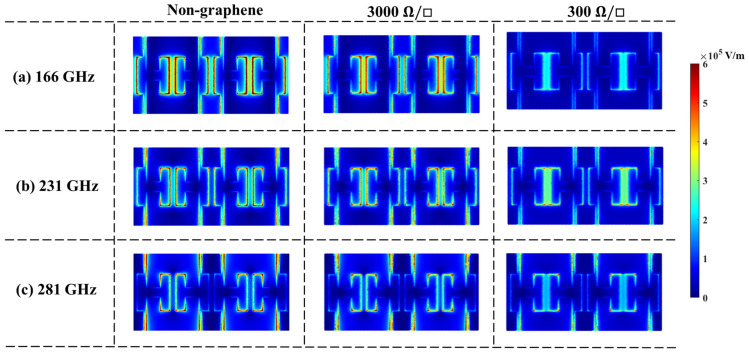
Simulated electric field distribution under non-graphene and added graphene with various sheet resistances at (**a**) 166 GHz, (**b**) 231 GHz, and (**c**) 281 GHz, respectively.

The simulated electric field distributions of TCMAs with various graphene sheet resistances are illustrated in Figure 6. For the non-graphene case, the dipoles can effectively capture the incident THz wave at the resonant frequency of 231 GHz. In contrast, the fields cannot be concentrated by the dipoles at 281 GHz and are confined between the bias lines at 166 GHz due to the plasmonic modes. When the graphene sheet resistance is set to *ρ_s_* = 3000 Ω/□, minimal differences are observed in both the dipole mode at 231 GHz and the plasmonic modes at 166 and 281 GHz. As the sheet resistance reduces to 300 Ω/□, the dipoles are effectively connected as an infinite current sheet, and the dipole mode gets much stronger at 166, 231, and 281 GHz, showing a much broader bandwidth.

### 2.3. Fabrication and Measurement

**Figure 7:**
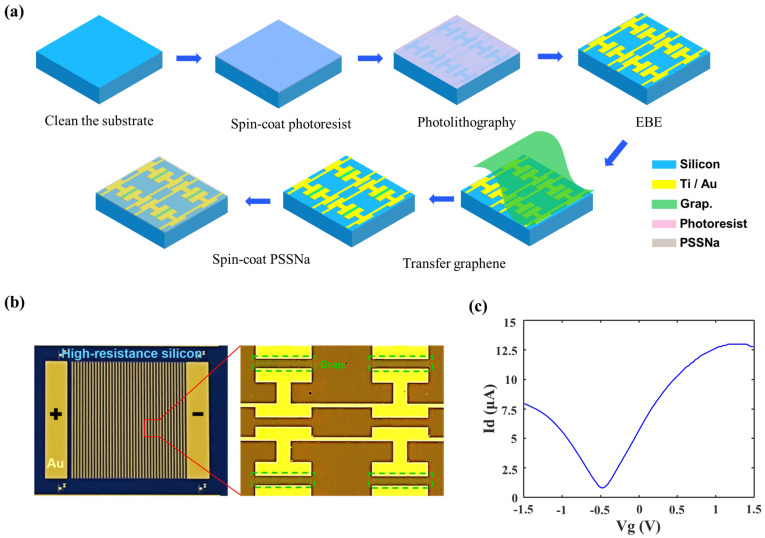
(**a**) Device fabrication process. (**b**) The image of the fabricated device. (**c**) The graphene Fermi–Dirac point DC test is conducted with a channel length of 300 μm and a width of 350 μm.

**Figure 8:**
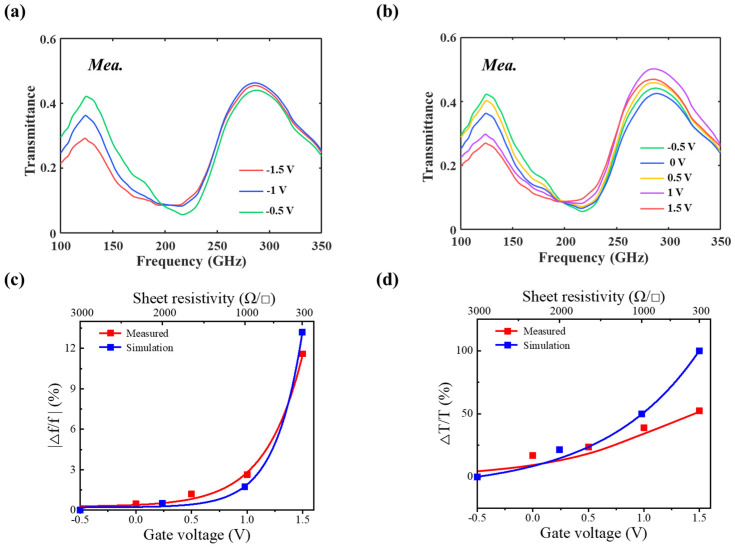
The measured transmittance of the fabricated device under gate bias sweeping (**a**) from −1.5 V to −0.5 V and (**b**) from −0.5 V to 1.5 V. (**c**,**d**) The measured and simulated modulation depths of resonant frequency and transmittance, respectively.

Figure 7a,b show the proposed TCDAs’ fabrication process and the fabricated device, where graphene is outlined with a green dashed line in the inset, and the positive and negative electrodes are fabricated to apply the bias voltage on graphene. The fabrication details can be found in Section 4. To precisely reveal the modulation capability of the graphene load, a graphene thin-film transistor is fabricated on the same chip with TCMAs, and its I–V curve is characterized, as shown in Figure 7c. The Dirac point of the graphene is obviously around −0.5 V, where the calculated graphene sheet resistance approximately equals 3000 Ω/□. Figure 8a,b show the transmittance of TCMAs under various bias voltages applied to graphene at room temperature, as measured using the Toptica frequency domain spectrometer (FDS). The resonant frequency of TCMAs increases from 200 to 219.4 GHz under a bias sweeping from −1.5 V to −0.5 V and decreases from 219.4 to 194 GHz under a bias sweeping from −0.5 V to 1.5 V. Concurrently, the corresponding transmittance decreases from 0.1 to 0.059 under a bias from −1.5 V to −0.5 V and increases from 0.059 to 0.09 under a bias from −0.5 V to 1.5 V. The measured maximum modulation depths of resonant frequency and transmittance are 11.6% and 52.5%, respectively, at 1.5 V. As shown in Figure 8c,d, the measured modulation depth of resonant frequency approximates the simulated data well, while the measured modulation depth of transmittance is smaller than the simulated counterparts. This discrepancy may be attributed to the random defects in the one-step transferred graphene, which could potentially hamper wave transmittance, thus resulting in lower transmittance values than anticipated.

## 3. Conclusions

In summary, a metal–graphene hybrid H-shaped TCMA was proposed with ultra-high permittivity at terahertz frequencies, and its graphene modulation was designed with the classic TCDA theory. The simulation indicates that graphene loads can achieve the remarkable modulation of both the real (up to 77.8%) and imaginary (up to 61%) parts of the effective permittivity. The simulated modulation depth of the resonant frequency is 13.2%, while the counterpart for transmittance reaches 100%. Experimental data reveal a maximum modulation depth of 11.6% for the resonant frequency and 52.5% for transmittance under a minimal gate bias of less than 1.5 V, showing good consistency with the simulation. Our study establishes a new approach for the active modulation of tightly coupled metamaterials.

## 4. Materials and Methods

### 4.1. Fabrication

The device was fabricated on a 200 μm thick, high-resistivity silicon substrate, which included 40 × 40 units. Conventional lithography was used to define the pattern. The positive photoresist used in this experiment was AZ5350. Following this, 5 nm titanium (Ti), as the adhesion layer, and 100 nm gold (Au) films were deposited with electron beam evaporation (EBE) and lifted off with acetone. Next, a piece of monolayer CVD graphene film coated with PMMA was transferred onto the pre-patterned substrate and patterned by conventional photolithography and the inductively coupled plasma (ICP) etching process. The samples were cleaned in acetone to remove the photoresist residuals.

### 4.2. Graphene Transfer

The monolayer CVD graphene coated with a layer of PMMA was purchased from Xianfeng Nano company (Nanjing, China). This graphene piece contained a supporting layer of paper below the graphene layer. Firstly, a piece of graphene was carefully cut from the whole graphene layer with a size of 12 × 12 mm^2^. Next, the prepared graphene was immersed in deionized water for one hour. The graphene layer floated on the water’s surface, and after one hour, it was detached from the paper due to the interaction with deionized water. The paper was removed from the deionized water before the graphene was transferred onto the meta-atom surface. Then, the silicon substrate was put into the deionized water. The graphene layer stuck to the surface of the meta-atom substrate by moving either the graphene or the substrate, ensuring no wrinkles appeared. A nitrogen gas gun was employed to remove residual deionized water and dry the substrate with graphene. Then, the substrate was put on a hotplate and annealed at 90 °C for 1 h, improving the adhesion between the graphene and substrate. Once the substrate cooled down at room temperature, it was immersed in acetone for 30 min to remove the PMMA on top of the graphene. The dried substrate with graphene transfer could be used for photolithography and ICP etching processing. As shown in the right picture in Figure 7b, the etched graphene covered the gap between H-shaped meta-atoms with a 1 μm overlap for good contact.

### 4.3. Ion Gel Preparation

PSSNa, D-sorbitol, glycerol, and DI water (with a weight ratio of 40, 10, 10, and 40%) were mixed with magnetic stirring. After stirring for 2 h at room temperature, the ion gel was spin-coated onto the metallic film with a spinning rate of 1000 rpm for 1 min. Before the experimental test, a 70 μm thick layer of PSSNa was spin-coated onto the metastructures containing graphene to form an electric double-layer capacitor (EDLC) and provide gate bias for graphene. Considering that PSSNa is a water-based ion gel, the DC bias in the experiment should usually be less than 1.5 V to prevent an electrochemical reaction, which may cause irreversible damage to PSSNa EDLC and thus the degraded modulation range for graphene conductivity.

### 4.4. THz Characterization

The transmittance of the fabricated devices was characterized using a Toptica TeraScan 1550 THz frequency-domain spectrometer (FDS) manufactured by TOPTICA Photonics (Munich, Germany) at room temperature. The incident THz wave was generated using two continuous-wave lasers with a differential frequency method and focused with a beam radius of approximately 1 mm in a four-mirror reflection system. Spectral resolution down to 10 MHz was achieved, with normalization to the water vapor absorption lines to ensure stability in humidity conditions. The THz wave was perpendicularly incident on the TCMA surface and polarized perpendicular to the gap. During the spectral test, the DC bias was applied to the graphene through two electrodes and PPSNa. The characterized absorption spectra could be displayed on the according software, and the transmission spectra were obtained by comparison between water absorption and the meta-atom spectrum. 

### 4.5. Simulation

The 3D models of the structures were simulated with master and slave boundaries, i.e., a kind of periodic boundary, in Ansys High-Frequency Structural Simulator (HFSS), which applies the finite element method (FEM) to the calculation and analysis. This S-matrix includes Floquet Port 1 situated on the top surface of the air box and Floquet Port 2 positioned on the bottom surface of the air box, as shown in Figure 2b.

## Data Availability

The data presented in this study are available on request from the first or corresponding author.
